# Digitale Gesundheitskompetenz und Wohlbefinden: Ergebnisse einer quantitativen Studie über Studierende in Österreich während der Coronapandemie

**DOI:** 10.1007/s11553-022-00954-0

**Published:** 2022-05-29

**Authors:** Franziska Reitegger, Michaela Wright, Jessica Berger, Barbara Gasteiger-Klicpera

**Affiliations:** 1 Institut für Bildungsforschung und PädagogInnenbildung, Arbeitsbereich Inklusive Bildung und Heilpädagogische Psychologie, Universität Graz, Graz, Österreich; 2 Forschungszentrum für Inklusive Bildung, Universität Graz, Graz, Österreich

**Keywords:** Studierende, Psychische Gesundheit, Angst, COVID-19, Protektive Faktoren, University students, Mental Health, Anxiety, COVID-19, Protective factors

## Abstract

**Hintergrund:**

Bisherige Befunde lassen darauf schließen, dass sich seit Beginn der Pandemie depressive und angstspezifische Symptome bei Studierenden verdoppelt haben. Digitale Gesundheitskompetenz kann hier als protektive Ressource zur Stärkung des Wohlbefindens fungieren.

**Ziel:**

Dieser Beitrag analysiert den Zusammenhang von digitaler Gesundheitskompetenz, dem sozioökonomischen Status sowie Wohlbefinden und Zukunftsangst bei Studierenden in Österreich.

**Methode:**

Mittels Online-Fragebogen wurden 480 Studierende österreichischer Hochschulen während der 2. Welle der Pandemie befragt. Es wurden soziodemographische Daten, Selbsteinschätzungen der Studierenden zum Wohlbefinden, zu Zukunftsängsten und zur digitalen Gesundheitskompetenz erhoben. Die Auswertung erfolgte mittels Varianz- und Regressionsanalysen.

**Ergebnisse:**

Etwa 50 % der Studierenden berichteten über ein geringes Wohlbefinden und deutliche Zukunftsängste. In Bezug auf die digitale Gesundheitskompetenz weist die Fähigkeit zur Beurteilung der Relevanz von Informationen den größten Zusammenhang mit dem Wohlbefinden auf. Ein höherer sozioökonomischer Status korrelierte sowohl mit einem höheren Wohlbefinden als auch mit niedrigeren Zukunftsängsten.

**Diskussion:**

Die Beurteilung der Relevanz von Informationen und die Herstellung des Bezugs zur eigenen Lebensrealität scheint ein wichtiger Faktor bei der Sicherung des Wohlbefindens zu sein. Individuelle Faktoren wie das Geschlecht oder das Studienprogramm sind für den Zusammenhang von Wohlbefinden und digitaler Gesundheitskompetenz von Relevanz.

## Abstract (en)

**Background:**

Previous findings suggest that depressive and anxiety-related symptoms have doubled among students since the beginning of the coronavirus pandemic. Digital health literacy can act as a protective resource to strengthen well-being.

**Objectives:**

This paper analyzes the relationship between digital health literacy, socioeconomic status, well-being and future-anxiety among students in Austria.

**Methods:**

A total of 480 students from Austrian higher education institutions were surveyed via online questionnaire during the second wave of the coronavirus pandemic. Sociodemographic data, students’ self-assessments of well-being, fears about the future, and digital health literacy were collected. Variance and regression analyses were used for the evaluation.

**Results:**

About 50% of the students reported low well-being and distinct fears about the future. Regarding digital health literacy, the ability to assess the relevance of information showed the highest correlation with well-being. A higher socioeconomic status correlated with higher well-being as well as lower fears about the future.

**Conclusions:**

The assessment of the relevance of information and connecting it with one’s own life reality seems to be an important factor in promoting well-being. Individual factors such as gender or the study program are relevant for the relationship between well-being and digital health literacy.

Bisher ist wenig über die Belastungen Studierender während der COVID-19-Pandemie („coronavirus disease 2019“) bekannt. Erste Befunde weisen darauf hin, dass das psychische Wohlbefinden abgenommen hat. Die digitale Gesundheitskompetenz kann hier als Schutzfaktor fungieren, es ist jedoch anzunehmen, dass diese vom Bildungsniveau und damit vom sozialen Status abhängig ist. Der vorliegende Beitrag analysiert, wie Studierende in Österreich mit den Herausforderungen der Pandemie umgehen, wie es um ihr Wohlbefinden steht und welche Rolle dabei die digitale Gesundheitskompetenz und der sozioökonomische Status einnehmen.

## Hintergrund und Fragestellung

### Wohlbefinden unter Studierenden während Corona

Die Coronapandemie veränderte den Lebensalltag der Studierenden und beeinträchtigt ihre Lebensqualität auf vielen Ebenen. Die soziale Isolation aufgrund der Schutzmaßnahmen zur Eindämmung der Pandemie erhöhte das Risiko für Depressionen und Angstzustände über alle Altersgruppen hinweg [3, 11, 14–16]. Diese Entwicklung lässt sich auch bei Studierenden feststellen, die aufgrund von erhöhter Unsicherheit und geringer sozialer Ressourcen eine besonders vulnerable Gruppe darstellen. Für sie erschwerten sich die Lebensbedingungen, da sie von ihrer Peergroup distanziert waren und in den ersten Semestern keine Gelegenheit hatten, sich in der neuen sozialen Situation an der Universität zu orientieren und neue soziale Kontakte aufzubauen. Zudem verschlechterte sich die Planbarkeit ihres Studiums und ihrer Zukunft [4]. Bereits vor der Pandemie wurde über steigende Zahlen von psychischen Erkrankungen bei Studierenden berichtet [7]. Damals gaben ca. 25 % der Studierenden an, nicht mit ihrer Lebenssituation zufrieden zu sein, 15,6 % berichteten über Depressionen und 17,4 % über Angststörungen [8]. Erste Befunde belegen nun eine erhebliche Verschlechterung ihrer psychischen Gesundheit. Studierende berichten über eine verstärkte Beeinträchtigung ihres Wohlbefindens (75 %) sowie über Einsamkeitsgefühle und Depressionen (41,8 %), aber auch von Angststörungen (20 %; [10]). Besonders deutlich sind Zukunftsängste, die durch die Unsicherheit der Situation und die wahrgenommene Bedrohung durch das Virus ausgelöst werden. Sie scheinen einen maßgeblichen Einfluss auf das Wohlbefinden zu haben [13].

### Protektive Faktoren

Betrachtet man die konkreten Veränderungen im Lebensalltag der Studierenden, so sind die Reduzierung sozialer Kontakte und die Konzentration auf digitale Lehr-/und Lernformate wohl die gravierendsten Veränderungen. Neu erworbene und verstärkte digitale Kompetenzen während der Coronapandemie können andererseits als wichtige Ressource hinsichtlich der individuellen Förderung der psychischen Gesundheit gesehen werden [1]. Wenn es um den Zusammenhang von digitalen Kompetenzen und Wohlbefinden geht, nimmt v. a. die digitale Gesundheitskompetenz einen zentralen Stellenwert ein. Eine hohe digitale Gesundheitskompetenz ist mit einem höheren Wohlbefinden bei Studierenden verbunden [4].

Das Konzept der Gesundheitskompetenz bezieht sich auf die Fähigkeit des Findens, Verstehens, Bewertens und Anwendens von gesundheitsbezogenen Informationen [17] und kann als digitale Gesundheitskompetenz auf digitale Informationen übertragen werden. Digitale Gesundheitskompetenz kann dementsprechend als zweidimensionales Konstrukt gesehen werden, bei dem es einerseits um die Fähigkeit der Nutzung digitaler Ressourcen geht, um auf Gesundheitsinformationen zugreifen zu können und andererseits, um kritische Informationskompetenz, die Fähigkeit, die Informationen sammeln, verstehen, bewerten und anwenden zu können [19, 22]. Dies schließt auch die Fähigkeit der kritischen Bewertung der Zuverlässigkeit und Relevanz von Informationen mit ein und ist besonders in Pandemiezeiten bedeutsam, da aufgrund sozialer Isolation keine direkten menschlichen Informationsquellen genutzt bzw. in Anspruch genommen werden können. Zudem können im Internet neue und aktuelle Erkenntnisse rascher verbreitet werden, was gerade in der derzeitigen Situation besonders wichtig ist. Daher gewinnt das Internet aufgrund der zunehmenden Verbreitung digitaler Angebote in der Gesundheitsversorgung und Risikoprävention an Bedeutung und wird als wichtigste Quelle gesehen, um zu Gesundheitsinformationen zu gelangen [12].

Neben den Befunden, die dafürsprechen, dass der digitalen Gesundheitskompetenz für die psychische Gesundheit besondere Bedeutung zukommt, gibt es Erkenntnisse, die darauf hinweisen, dass die digitale Gesundheitskompetenz mit dem sozioökonomischen Status zusammenhängt [1]. Deshalb sollte dieser bei der Betrachtung des Zusammenhangs zwischen dem Wohlbefinden und digitaler Gesundheitskompetenz mit berücksichtigt werden.

Um zu untersuchen, inwiefern ein Zusammenhang zwischen der digitalen Gesundheitskompetenz, dem sozioökonomischen Status und dem Wohlbefinden bei Österreichs Studierenden besteht, adressiert dieser Beitrag folgende Fragestellungen:

1. Wie ausgeprägt ist das Wohlbefinden von Studierenden in Österreich während der Coronapandemie?
2. Inwiefern besteht ein Zusammenhang zwischen dem Wohlbefinden und der digitalen Gesundheitskompetenz bei Studierenden in Österreich?
3. Inwiefern besteht ein Zusammenhang zwischen dem sozioökonomischen Status, der digitalen Gesundheitskompetenz und dem Wohlbefinden bei Studierenden in Österreich?

## Methodik

### Stichprobe

Wie in Tab. 1 ersichtlich, bestand die Untersuchungsstichprobe aus 480 Studierenden, die zum Zeitpunkt der Erhebung an einer österreichischen Hochschule studierten. Diese waren im Alter von 18–72 (Durchschnittsalter: 24,5) Jahren und 79,8 % fühlten sich dem weiblichen, 19,4 % dem männlichen und 0,8 % einem anderen Geschlecht zugehörig. Ähnlich den allgemeinen Studierendenzahlen befand sich die Mehrheit der Teilnehmenden im Bachelorstudium (64,8 %), während nur etwas mehr als ein Viertel (26 %) ein Masterstudium belegte. Der geringste Anteil bestand aus Studierenden aus sonstigen Studiengängen (z. B. Promotion) mit 9,2 %.

**Tab. 1:** Charakterisierung der Gesamtstichprobe (*n* = 480)

Geschlecht	Alter (Jahre) MW (SD)	Hochschule				Studienprogramm			Gesamt *n* (%)
		Uni	FH	PH	Priv. Uni	Bachelor	Master	Sonstiges (PhD)	
Männlich	25,5 (6,43)	84	2	3	4	60	21	12	93 (19,4)
Weiblich	24,2 (6,17)	343	3	18	19	248	104	31	383 (79,8)
Divers	27,3 (5,19)	4	0	0	0	3	0	1	4 (0,8)
Gesamt	24,5 (6,23)	431	5	21	23	311	125	44	*480 (100)*

*MW* Mittelwert, *SD* Standardabweichung

### Studiendesign

Die Studie wurde im Rahmen des internationalen Netzwerks COVID-HL (COVID-Health Literacy, covid-hl.eu) zur Erforschung von Gesundheit und Gesundheitskompetenz in Zeiten der Coronapandemie mit dem Fokus auf Studierende an österreichischen Hochschulen durchgeführt. Der im Netzwerk entwickelte Fragebogen [6] wurde an die in Österreich vorherrschenden Gegebenheiten (z. B. bei der Auswahl der Hochschulen) angepasst, in die deutsche Sprache übersetzt und mittels „lime survey“ als Online-Fragebogen aufbereitet, bevor er durch die Österreichischen Hochschüler*innenschaften an die Studierenden der unterschiedlichen Hochschulen per E‑Mail vergesandt wurde. Die erste Kontaktaufnahme erfolgte per E‑Mail am 11.06.2021, am 15.07.2021 folgte eine kurze Erinnerung mit der erneuten Bitte um Unterstützung bei der Verbreitung des Online-Fragebogens. Auf diese Weise sollten möglichst viele Studierende an den insgesamt 22 Universitäten, 21 Fachhochschulen, 14 Pädagogischen Hochschulen und 16 Privatuniversitäten in Österreich erreicht werden. Der Zeitraum der Umfrage erstreckte sich von Juni bis September 2021. Zu diesem Zeitpunkt befand sich Österreich in der zweiten Welle der COVID-19-Pandemie („coronavirus disease 2019“). Daher konnten die Studierenden nur eingeschränkt und unter Einhaltung der von der Regierung vorgeschriebenen Maßnahmen, die von den Universitäten unterschiedlich umgesetzt wurden, ihrem Studium nachgehen. An den meisten Universitäten wurde überwiegend „distance learning“ umgesetzt.

### Untersuchungsinstrumente

#### Soziodemographische Informationen

Zu Beginn des Fragebogens wurden die Studierenden zu ihrem Geschlecht (weiblich, männlich, anderes Geschlecht), Alter, der besuchten Hochschulform (Universität, Fachhochschule, Pädagogische Hochschule, Private Universität) sowie dem Studienprogramm (Bachelor, Master, sonstige z. B. Promotion) befragt. Zusätzlich wurde der subjektive soziale Status mittels der deutschen Version der MacArthur-Scale [9] abgefragt. Hier nehmen die Befragten eine Selbsteinschätzung des eigenen sozialen Status im Vergleich zu ihren Mitbürger*innen auf einer zehnsprossigen Leiter vor (1 = niedrig; 10 = hoch).

#### Digitale Gesundheitskompetenz

Die digitale Gesundheitskompetenz der Studierenden wurde mittels 4 vom Netzwerk adaptierten Subskalen [6] des Digital-health-literacy-Fragebogens von Van der Vaart [19] erhoben. Diese Subskalen umfassen die Selbsteinschätzung der Schwierigkeitsgrade in den Bereichen 1. Informationssuche (i.O. „information searching“; z. B. wenn Sie im Internet nach Informationen über das Coronavirus oder verwandte Themen suchen, wie einfach oder schwierig ist es für Sie, genau die Informationen zu finden, nach denen Sie suchen?) 2. Erstellung eigener Inhalte/Beiträge (i.O. „adding self-generated content“; z. B. wenn Sie eine Nachricht über das Coronavirus oder verwandte Themen schreiben, wie leicht oder schwer ist es für Sie, Ihre Meinung, Gedanken oder Gefühle schriftlich auszudrücken?) 3. Bewertung der Zuverlässigkeit und Vertrauenswürdigkeit (i.O. „evaluating reliability“; z. B. wenn Sie im Internet nach Informationen über das Coronavirus oder verwandte Themen suchen, wie einfach oder schwierig ist es für Sie zu entscheiden, ob die Informationen zuverlässig sind oder nicht?) sowie 4. Einschätzung der Relevanz der gefundenen Informationen (i.O. „determining relevance“; z. B. wenn Sie im Internet nach Informationen über das Coronavirus oder verwandte Themen suchen, wie einfach oder schwierig ist es für Sie zu entscheiden, ob die Informationen, die Sie gefunden haben, auf Sie zutreffen?). Jede Subskala besteht aus 3 Items, die auf einer vierstufigen Likert-Skala beantwortet werden (1 = häufig bis 4 = nie). Es zeigte sich eine ausreichende bis gute Reliabilität der 4 Subskalen (α = 0,79–0,84). Die 5. Subskala des Originalfragebogens (Umgang mit personenbezogenen Informationen und Datenschutz, i.O. „protecting privacy“) wurde aufgrund ihrer niedrigen Reliabilität (α = 0,52) ausgeschlossen.

#### Wohlbefinden

Das Wohlbefinden der Studierenden wurde mit dem WHO-5-Wohlbefindensindex [2, 18, 20] erfasst. Dieser Kurzfragebogen misst das subjektive Wohlbefinden mittels fünf Aussagen zur Häufigkeit von positiven Erfahrungen in den letzten Monaten (z. B.: Während der letzten Monate war ich froh und guter Laune), die auf einer sechsstufigen Likert-Skala (0 = nie, 1 = ab und zu, 2 = etwas weniger als die Hälfte der Zeit, 3 = etwas mehr als die Hälfte der Zeit, 4 = meistens, 5 = die ganze Zeit) eingeschätzt werden. Der Summenscore der Skala liegt zwischen 0 (niedriges Wohlbefinden) und 100 (optimales Wohlbefinden) und es wird ein Cut-off-Score von ≤ 50 beim Screening für Depressionen empfohlen. In der vorliegenden Studie betrug das Cronbachʼs Alpha der Skala 0,88.

#### Zukunftsangst

Um Zukunftsängste zu messen, wurde eine Kurzversion der Future-Anxiety Scale, die sog. Dark-Future Scale [21] eingesetzt. Diese umfasst 5 Items zu verschiedenen Formen von Zukunftsängsten (z. B. Ich habe Angst, dass sich mein Leben in Zukunft zum Schlechten verändern wird), die auf einer siebenstufigen Likert-Skala (1 = entschieden falsch bis 7 = entschieden wahr) beantwortet werden. Die Skala verfügt über gute psychometrische Eigenschaften, das Cronbachʼs Alpha betrug in der vorliegenden Studie 0,79.

### Statistische Analysen

Die statistischen Berechnungen wurden mit IBM SPSS Statistic 27 (IBM Corp., Armonk, NY, USA) durchgeführt. Mittelwertsunterschiede wurden mit Multivariaten Varianzanalysen geprüft; um Zusammenhangshypothesen zu analysieren, wurden Korrelationen nach Pearson berechnet.

## Ergebnisse

### Wohlbefinden

Im Durchschnitt berichteten die Studierenden von einem eher geringen Wohlbefinden (MW = 42,68, SD = 22,31). Unter Anwendung des empfohlenen Cut-off-Scores von ≤ 50 berichteten 49,8 % der Studierenden über ein niedriges Wohlbefinden bzw. über Anzeichen einer Depression. Davon fällt ein Anteil von 27,1 % der Studiereden unter den Cut-off-Score von 28, der einen noch restriktiveren Schwellenwert beim Screening von Depression darstellt [18]. Eine detaillierte Darstellung der Mittelwerte nach Geschlecht, Hochschultyp und Studienprogramm befindet sich in Tab. 2. Weder nach Geschlecht (*p* =0,22) noch nach Hochschultyp (*p* =0,07) sind signifikante Unterschiede zwischen den Gruppen erkennbar. Ein signifikanter Unterschied ergab sich jedoch hinsichtlich des jeweiligen Studienprogramms (F_2,386_ = 6,849, *p* =0,001, η^2^ = 0,034). Bachelorstudierende wiesen ein geringeres Wohlbefinden im Vergleich zu Studierenden anderer Studiengänge auf.

**Tab. 2:** Wohlbefinden und Zukunftsangst nach Geschlecht, Hochschule und Studienprogramm

		Wohlbefinden	Zukunftsangst
		MW (SD)	MW (SD)
Geschlecht	Männlich	44,05 (24,61)	20,29 (6,74)
	Weiblich	42,54 (21,71)	21,08 (6,75)
Hochschule	Universität	42,06 (22,12)	21,07 (6,78)
	Fachhochschule	40,00 (33,11)	19,40 (8,56)
	Pädagogische Hochschule	57,25 (18,75)	18,80 (6,49)
	Private Universität	42,67 (23,24)	21,90 (5,79)
Studienprogramm	Bachelor	40,23 (21,48)	21,95 (6,46)
	Master	44,58 (23,35)	19,13 (6,94)
	Sonstiges (PhD)	54,67 (20,92)	19,61 (6,92)

*MW* Mittelwert, *SD* Standardabweichung

### Zukunftsangst

Im Durchschnitt berichteten die Studierenden von eher hohen Zukunftsängsten (MW = 20,98, SD = 6,74; Tab. 2). Bei Gruppierung der Scores in niedrig (1–16), eher niedrig (17–21), eher hoch (22–26) und hoch (27–35) zeigt sich, dass 22,3 % der Studierenden von eher hohen und 20,6 % von hohen Zukunftsängsten berichten. Differenziert betrachtet ergeben sich, wie bereits beim Wohlbefinden nach Geschlecht und Hochschultyp, keine signifikanten Unterschiede, jedoch auch hier nach Studienprogramm, wonach Bachelorstudierende im Vergleich zu anderen Studienprogrammen häufiger von Zukunftsängsten berichteten (F_2,438_ = 8,470, *p* = 0,000, η^2^ = 0,037).

### Digitale Gesundheitskompetenz und Wohlbefinden

Wie in Tab. 3 ersichtlich, konnte ein hochsignifikant positiver Zusammenhang zwischen der Relevanzbeurteilung und dem Wohlbefinden nachgewiesen werden. Die anderen Komponenten der digitalen Gesundheitskompetenz wiesen keinen signifikanten Zusammenhang mit dem Wohlbefinden auf. Allerdings sind die Interkorrelationen der einzelnen Skalen der digitalen Gesundheitskompetenz relativ hoch. Sie sind somit nicht ganz unabhängig voneinander.

**Tab. 3:** Korrelationen Wohlbefindensindikatoren und digitale Gesundheitskompetenz (*n* = 480)

Skala	Korrelationen				
	1	2	3	4	5
1 Wohlbefinden	–	–	–	–	–
2 Informationssuche	0,061	–	–	–	–
3 Erstellung eigener Inhalte	0,063	0,325^b^	–	–	–
4 Bewertung der Zuverlässigkeit	0,044	0,538^b^	0,339^b^	–	–
5 Einschätzung der Relevanz	0,139^a^	0,510^b^	0,436^b^	0,550^b^	–
6 Zukunftsangst	−0,452^b^	−0,150^b^	−0,136^a^	−0,126^a^	−0,207^b^

^a^Pearson-Korrelation auf Niveau 0,05 signifikant

^b^Pearson-Korrelation auf Niveau 0,01 signifikant

Anders verhält es sich in Bezug auf Zukunftsängste, die mit allen Skalen der digitalen Gesundheitskompetenz signifikant negativ korrelierten. Ein geringeres Ausmaß an Zukunftsängsten hängt demnach mit höheren digitalen Gesundheitskompetenzen in allen Bereichen zusammen. Ein besonders deutlicher negativer Zusammenhang zeigt sich auch hier mit der Skala Einschätzung der Relevanz.

### Sozioökonomischer Status und Wohlbefinden

**Abb. 1:**
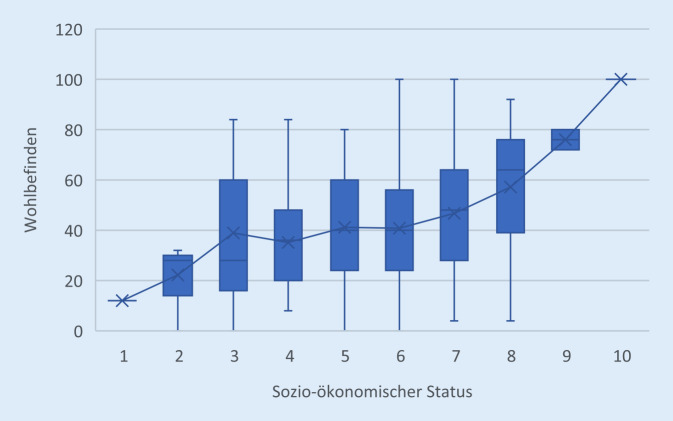
Wohlbefinden nach sozioökonomischem Status

**Abb. 2:**
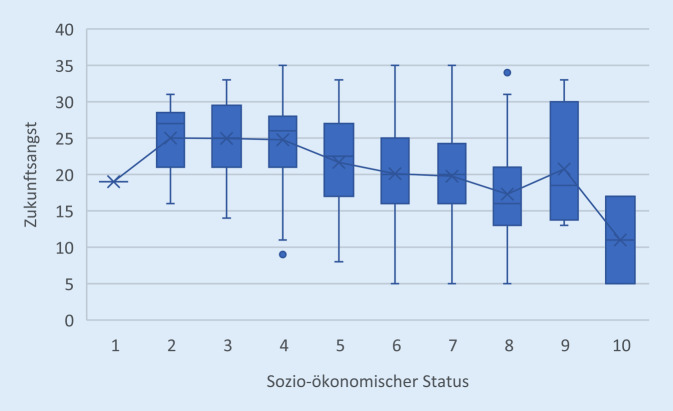
Zukunftsangst nach sozioökonomischem Status

Wie in Abb. 1 ersichtlich, nimmt das Wohlbefindens mit zunehmendem selbsteingeschätztem sozioökonomischem Status zu. Auch die multivariate Varianzanalyse bestätigt den deutlich positiven Zusammenhang zwischen der subjektiven Einschätzung des sozialen Status und dem Wohlbefinden (F_9,388_ = 5,570, *p* =0,000, η^2^ = 0,117). Ähnlich verhält es sich mit der Zukunftsangst (Abb. 2). Mit steigendem sozioökonomischem Status ist eine signifikante Abnahme der zukunftsbezogenen Ängste zu verzeichnen (F_9,438_ = 6,250, *p* = 0,000, η^2^ = 0,116).

### Sozioökonomischer Status, digitale Gesundheitskompetenz und Wohlbefinden

Bei Analyse des Zusammenhangs zwischen dem sozioökonomischen Status und den einzelnen Subskalen der digitalen Gesundheitskompetenz konnte ein signifikanter Zusammenhang mit der Skala Informationssuche identifiziert werden (F_9,342_ = 2,074, *p* = 0,031, η^2^ = 0,053). Studierende mit höherem sozialem Status fällt es demnach leichter, im Internet nach Informationen zu suchen.

Mittels weiterer multivariater Analysen konnten keine geschlechtsspezifischen Unterschiede identifiziert werden, jedoch konnten signifikante Interaktionseffekte von Geschlecht und Wohlbefinden in den Bereichen Erstellung eigener Beiträge (F_1,298_ = 3,919 *p* = 0,049, η^2^ = 0,013) und Überprüfung der Zuverlässigkeit (F_1,298_ = 7,328, *p* = 0,007, η^2^ = 0,024) festgestellt werden. Demnach finden es weibliche Studierende, die über ein gutes Wohlbefinden berichten einfacher, die Zuverlässigkeit von Informationen zu beurteilen sowie eigene Beiträge zu verfassen, als männliche Studierende. Umgekehrt finden es männliche Studierende mit geringem Wohlbefinden einfacher, die Zuverlässigkeit von Informationen zu beurteilen und eigene Beiträge zu verfassen als weibliche.

### Veränderungen im Zusammenhang von sozioökonomischen Status und Wohlbefinden bei Berücksichtigung der digitalen Gesundheitskompetenz

Um zu überprüfen, ob sich die Stärke des Zusammenhangs des sozioökonomischen Status mit dem Wohlbefinden verändert, wenn die digitale Gesundheitskompetenz miteinbezogen wird, wurde eine schrittweise lineare Regression berechnet.

Das erste Regressionsmodell (Modell 1) zeigt den Zusammenhang der Variable sozioökonomischer Status mit dem Wohlbefinden, der mit einem Wert von β = 0,285 deutlich und statistisch signifikant (*p* < 0,01) ist. Darüber hinaus wird ersichtlich, dass über den sozioökonomischen Status 7,8 % der Varianz des Wohlbefindens aufgeklärt werden können.

Bei Betrachtung von Modell 2, bei dem die 4 Skalen zur digitalen Gesundheitskompetenz mit einbezogen wurden, zeigt sich, dass die Varianzaufklärung nur marginal ansteigt. Nun können 8,1 % der Varianz aufgeklärt werden. Gleichzeitig reduziert sich die Bedeutung des sozioökonomischen Status (β = 0,280, *p* < 0,01). Der Betakoeffizient der Einschätzung der Relevanz ist zwar nicht-signifikant (β = 0,138, *p* = 0,06), kann jedoch aufgrund seiner Höhe als potenziell wichtiger Faktor für das Wohlbefinden neben dem sozialen Status gesehen werden (Tab. 4).

**Tab. 4:** Lineare Regressionen zum Zusammenhang des sozioökonomischen Status mit dem Wohlbefinden (Modell 1) bzw. des sozioökonomischen Status und der digitalen Gesundheitskompetenz mit dem Wohlbefinden (Modell 2)

	Modell 1		Modell 2	
	*B (SD)*	*Beta*	*B (SD)*	*Beta*
Konstante	17,917^a^ (5,071)	–	8.427 (8,311)	–
SES	4.408 (0,855)	0,285^a^	4.329 (0,869)	0,280^a^
Informationssuche	–	–	−0,524 (0,732)	−0,050
Erstellung eigener Inhalte	–	–	0,298 (0,710)	0,026
Bewertung der Zuverlässigkeit	–	–	−0,317 (0,735)	−0,031
Einschätzung der Relevanz	–	–	1,624 (0,860)	0,138
–	Korr. R^2^ = 0,078 F = 26,612^a^	–	Korr. R^2^ = 0,081 F = 6,318^a^	–

^a^*p* < 0,01

## Zusammenfassung und Diskussion

Ziel der vorliegenden Studie war die Untersuchung des Wohlbefindens von Studierenden in Zeiten der COVID-19-Pandemie und die Analyse der Frage, ob die digitale Gesundheitskompetenz der Studierenden einen Beitrag zu ihrem Wohlbefinden leisten kann. Im Zusammenhang mit digitaler Gesundheitskompetenz wurde auch die Frage des sozioökonomischen Status der Studierenden analysiert.

Die Ergebnisse zeigen zunächst, dass fast 50 % der Studierenden und somit ca. 12 % mehr als in vergleichbaren Studien aus Deutschland [5] sowohl über ein geringes Wohlbefinden als auch über hohe Zukunftsängste berichten. Diesbezüglich sind im Gegensatz zu Ergebnissen aus anderen Ländern [5] keine geschlechtsspezifischen Unterschiede zu erkennen, jedoch berichten v. a. Bachelorstudierende über ein geringeres Wohlbefinden und stärkere Zukunftsangst. Es erscheint bemerkenswert, dass gerade jene, die noch weniger lang an der Universität studieren, durch die sozialen Restriktionen und das „distance learning“ stärker betroffen sind. Wahrscheinlich gelingt es Studierenden, die schon länger an der Hochschule lernen besser, sich an die neuen Bedingungen anzupassen und sich weiterhin gut an der Hochschule und im Studium zurechtzufinden.

In Bezug auf die digitale Gesundheitskompetenz konnte festgestellt werden, dass diese nicht in ihrer Gesamtheit, sondern lediglich in einzelnen Subbereichen Zusammenhänge mit dem Wohlbefinden aufweist. Konkret berichteten jene Studierenden, die die Schwierigkeit der Relevanzeinschätzung von Informationen im Internet als niedrig betrachteten, von einem signifikant besseren Wohlbefinden. Die Fähigkeit, aus der Fülle an digitalen Gesundheitsinformationen jene zu identifizieren, die für das eigene Leben relevant sind, kann demnach ein wichtiger protektiver Faktor sein. Bei Betrachtung des Zusammenhangs von digitaler Gesundheitskompetenz und Zukunftsängsten zeigte sich deutlich, dass höhere Gesundheitskompetenz in allen Teilbereichen mit weniger Zukunftsängsten verbunden ist, was dadurch erklärt werden kann, dass die Informationsbeschaffung zu gesundheitsrelevanten Themen und ein kompetenter Umgang mit den gefundenen Informationen Sicherheit und Vertrauen vermitteln und dadurch Zukunftsängste verringern kann.

Der sozioökonomische Status konnte in dieser Untersuchung als deutlicher Einflussfaktor auf das individuelle Wohlbefinden der Studierenden identifiziert werden, was sich mit bisherigen internationalen Ergebnissen deckt [1, 5]. Mit zunehmend höher eingeschätztem sozialem Status wurde von einem höheren Wohlbefinden und geringeren Zukunftsängsten berichtet. Ähnlich wie bei bisherigen Untersuchungen [1] zeigten weitere Analysen, dass sich dieser Zusammenhang auch unter Hinzuziehung der digitalen Gesundheitskompetenz nicht maßgeblich verringerte, weshalb bei Ansätzen zur Förderung dieser Kompetenzen das sozioökonomische Umfeld der Studierenden Berücksichtigung finden sollte [1].

Studierenden mit höherem sozialem Status fällt es leichter, im Internet nach Informationen zu suchen. Zudem wurden hier deutliche Geschlechtsunterschiede beobachtet. Weibliche Studierende, die über ein gutes Wohlbefinden berichten, verfügen über eine höhere digitale Gesundheitskompetenz in der Einschätzung der Zuverlässigkeit von Informationen sowie im Verfassen eigener Beiträge, als männliche Studierende. Beides wiederum fällt männlichen Studierenden mit geringem Wohlbefinden leichter.

Zusammenfassend zeichnet sich eine negative Entwicklung des psychischen Wohlbefindens von Studierenden ab. Das Potenzial der digitalen Gesundheitskompetenz als protektive Ressource konnte teilweise bestätigt werden, was die Relevanz weiterer Forschung in diesem Bereich verdeutlicht. Individuelle Faktoren wie der soziale Status sind hier ein relevanter Faktor, da sich herausgestellt hat, dass diesem eine maßgebliche Rolle hinsichtlich des Wohlbefindens zuzuschreiben ist. Dieser Zusammenhang zwischen dem Wohlbefinden und selbsteingeschätzten sozialen Status zeigte sich bei österreichischen Studierenden unabhängig von der Ausprägung ihrer digitalen Gesundheitskompetenz und konnte dementsprechend auch nicht durch diese verringert werden.

## Fazit für die Praxis

- Fast die Hälfte der untersuchten Studierenden in Österreich berichteten über ein geringes Wohlbefinden und hohe Zukunftsängste.
- Einzelne Komponenten der digitalen Gesundheitskompetenz konnten als protektive Ressource zur Förderung des Wohlbefindens bestätigt werden, wobei auch geschlechtsspezifische Unterschiede deutlich wurden. Die Kompetenz, Informationen hinsichtlich ihrer Relevanz für das eigene Leben zu beurteilen, scheint das größte Potenzial zur Stärkung des Wohlbefindens aufzuweisen.
- Der sozioökonomische Status weist einen deutlichen Zusammenhang mit dem Wohlbefinden auf.
- Der Zusammenhang zwischen dem sozialen Status und dem Wohlbefinden kann durch die digitale Gesundheitskompetenz verringert werden.
- Zukünftige Studien, die den Zusammenhang zwischen digitaler Gesundheitskompetenz und psychischen Variablen untersuchen, sollten individuelle Faktoren wie den sozioökonomischen Status, das Geschlecht und das Studienprogramm berücksichtigen.
